# Semantic Contingency of Maternal Verbal Input Directed at Very Preterm and Full-Term Children

**DOI:** 10.3389/fpsyg.2022.800568

**Published:** 2022-02-17

**Authors:** Nicoletta Salerni, Chiara Suttora

**Affiliations:** ^1^Department of Psychology, University of Milano - Bicocca, Milano, Italy; ^2^Department of Psychology “Renzo Canestrari”, University of Bologna, Bologna, Italy

**Keywords:** very preterm children, language development, responsiveness, maternal semantically contingent input, child-directed speech

## Abstract

Several studies have testified to the importance of a responsive linguistic input for children’s language acquisition and development. In particular, maternal use of expansions, imitations, interpretations, and labels has been shown to promote both children’s language comprehension and production. From this perspective, the present study examined the semantically contingent linguistic input addressed to very preterm children’s comparing it to that directed to full-term children observed during a semi-structured play session when the children were 24 months of age. The relationships between maternal contingent utterances and children’s communicative repertoires were also investigated. The main results showed that mothers of full-term children produced a higher proportion of semantically contingent utterances than those of very preterm children; moreover, this variable was associated with children’s more advanced communicative-linguistic outcomes. Overall, this study supports the interdependence between mothers’ use of certain linguistic strategies and children’s communicative-linguistic repertoire, extending this evidence to children born very preterm and suggesting the importance of considering the semantic contingency aspect of child-directed speech to support the communicative and linguistic development of these children.

## Introduction

### Child-Directed Speech: the Relevance of Maternal Input Contingency in Children’s Communicative Development

It is now widely accepted that the linguistic input young children are exposed to contribute to influencing both their language acquisition and development.

In this area of investigation, many studies focused on the quantitative aspects of child-directed speech (CDS) showing that one source of variability in language growth is represented by different amount of exposure to parents’ speech directly addressed to the infant (Huttenlocher et al., 1991, 2010; Hoff, 2003; Weisleder and Fernald, 2013).

Nonetheless, other empirical evidence pointed out that the quality of CDS plays an equally crucial role in fostering the development of early language skills. As recently argued by Rowe and Snow (2020) in their review on language input, three partially overlapping dimensions can be identified as facilitating language learning at different developmental stages, referring, respectively, to interactional features (i.e., responsiveness, shared attention, and discussion of child interest), linguistic aspects (i.e., phonological, lexical and grammatical levels of complexity, and redundancy), and conceptual content (i.e., topics of conversation).

In this regard, the social-interactionist perspective states that language learning is made easier if the content of the adult’s CDS corresponds to the child’s own processing mechanisms, namely, if it is responsive (Girolametto et al., 2002). Particularly, during the vocabulary development stage and up to the onset of the first word combinations, is well documented that maternal responsive behaviors, defined as contingent, appropriate, and prompt responses to a child’s communicative initiations (Bornstein et al., 2008), can be considered a valid predictor of children’s achievement of several language milestones (Tamis-LeMonda et al., 2001).

Although verbal responsiveness is considered a multidimensional construct susceptible to various operationalizations (McGillion et al., 2013), one aspect that has been focal in several studies is semantic contingency, in which the conversational partner verbally responds to the child’s focus of utterances or attention (Harkness, 1988). In other words, semantic contingency may be broadly defined as the maintenance, by the adult, of the communicative exchange topic, or interest, promoted by the child, while respecting conversational turn taking and providing verbal information specifically related to it. In this sense, the linguistic input that is both conversationally (temporally connected) and conceptually related to children’s utterances and actions (Rowe and Snow, 2020) is thought to increase the saliency of the input itself and the likelihood that words will be bound to real-world referents, as it reduces the cognitive loads the child needs to process linguistic information (McGillion et al., 2013; Tamis-LeMonda et al., 2014) and fosters the associations among language content, form, and use (Girolametto et al., 1999).

Different caregivers’ linguistic behaviors are contingent to the child’s attentional focus and topic of conversation, incorporating them into one’s own immediately following turn, such as the labeling of objects or events to which the child is paying attention to, the imitation of his/her verbal productions, the expansion of the child’s verbal initiatives, and the interpretation of child’s prelinguistic utterances (Conti-Ramsden, 1990; Girolametto et al., 1999). In other words, during interactive exchanges, the adult adopts a series of pragmatic strategies, sometimes referred as recasts, to scaffold child’s language production, by providing information about words meaning and use, correcting and reformulating incomplete or ungrammatical utterances, or repeating and extending them to encourage the production of more complex linguistic units (Clarke et al., 2017).

All of these strategies adopted by the caregiver—which are also aimed to ensure mutual involvement and active participation of the child in interaction through fine-tuning to his/her language level (Cross, 1977; Snow, 1989; Sokolov, 1993)—support children’s transition from prelinguistic to linguistic communication and promote accelerated language development, as demonstrated by several studies focusing on different lexical and morphosyntactic aspects, including vocabulary size, early vocabulary composition, word type growth, spontaneous productions of newly acquired semantic relations, acquisition of grammatical morphemes, and syntactic structures (Scherer and Olswang, 1984; Tomasello and Farrar, 1986; Farrar, 1990, 1992; Pine, 1994; Nelson et al., 1996; Tamis-LeMonda et al., 2001; McGillion et al., 2013; Taumoepeau, 2016).

At the same time, the importance of such input in promoting language development has also been highlighted in children with language delays or difficulties. In this regard, Girolametto and colleagues (Girolametto et al., 1999) pointed out that maternal responsive behaviors classified as expansions and imitations represented the best predictors of children’s expressive language outcomes (i.e., number of children’s utterances, number of different words, vocabulary size, and word combinations) in a group of male late talkers observed longitudinally. In line with these findings, in a later study, Girolametto et al. (2002) found that mothers’ semantically contingent language measures (i.e., imitations, interpretations, and expansions) were related to higher levels of verbal productivity in children exhibiting delays in expressive vocabulary development but age-appropriate cognitive and receptive language skills. More recently, a study aimed at determining, in a community-based sample of slow-to-talk toddlers, the extent to which specific maternal responsive behaviors at 24 months predict child language both concurrently and at 36 months (Levickis et al., 2014) showed that expansions, imitations, and responsive questions were strongly associated with better receptive and expressive language outcomes at both ages; moreover, expansions were the only maternal linguistic behavior that predicted language improvement by the end of children’s second year of life. The emphasis placed on expansions and labeling in predicting later language development is also confirmed in a study that points out that these parental responsive utterances, in addition to child’s earlier language skills, increase the ability to predict language outcomes at age 4 in a sample of children who are late to talk (Levickis et al., 2018).

### Maternal Verbal Input Directed to Children Born Preterm

Overall, empirical evidence seems to support that some of the variability observed in language abilities of both typically and atypically developing children can be explained by differences in features of language input, including responsiveness to children’s early focus of attention and communicative attempts.

For this reason, it may be relevant to investigate these characteristics of maternal input also in children born preterm since, from the early stages of development, they exhibit delays that tend to persist over time, with cascading effects on more sophisticated skills developing later in their life (De Schuymer et al., 2011). A large body of literature indicates the negative impact of premature birth on the development of language abilities, especially in children born extremely and very preterm (i.e., those born, respectively, before 28 and between 28 and 32 weeks of gestation). Studies involving very preterm children found that they show a slower acquisition in both word comprehension and production with an increasing divergence, with respect to full-terms, from 12 to 24 months (Sansavini et al., 2011); moreover, at 2 years of age, a considerable proportion of them do not produce word combinations or are characterized by a small expressive vocabulary, below the 10th percentile on the MB-CDI questionnaire (Sentenac et al., 2020). Similarly, some evidence suggests that even low-risk preterm score significantly lower than term infants on the BSID-III language scales at 24 months, considering both corrected and chronological age (Ionio et al., 2016), and that their language development appears suboptimal compared to that of their full-term peers, performing less favorably even as young as 2 years of age (Nepomnyaschy et al., 2012).

Although the difficulties observed in these children appear related to biologically determined factors, part of the observed variability in language skills can also be associated with external, environmental variables (Howard et al., 2011; Wild et al., 2013). In this area of investigation, studies addressing the potential role of caregiver input provided to preterm children do not seem to be many, thus highlighting a gap in the research aimed at exploring the interrelationships between the abovementioned factor and language skills in this population. Moreover, much of this research has primarily focused on structural-linguistic and/or quantitative aspects of maternal verbal input. For instance, some authors found that during the first months of life, mothers of premature infants, in comparison with those of term infants, follow significantly more frequently their infants’ non-cry vocalizations with an utterance directed at the child and initiate conversational turns more often (Reissland and Stephenson, 1999); nevertheless, similar quantities of words and utterances produced have been observed in the two groups of mothers, and no differences have been detected regarding lexical and syntactical complexity of linguistic input directed to preterm and full-term children (Salerni et al., 2007), even when considering a sample of low-risk preterm and full-term children with language delay (Suttora et al., 2020). In addition, findings suggested that changes over time in the structural characteristics of language input directed to preterm infants are also substantially similar to those described in the literature concerning typically developing infants: major increases in both lexical complexity and productivity were observed in the transition from 6 months to the second year, whereas the syntactic complexity of maternal speech, measured in terms of mean length of utterance, showed a significant increase during the second year of life (Suttora and Salerni, 2011).

As regards the functional characteristics of linguistic input, some studies highlighted that mothers of preterm children produced more directive and controlling utterances than mothers of term children (Menyuk et al., 2014), although others failed to detect such differences from 6 months of age onward (Hebert et al., 2004; Salerni et al., 2007). Despite these partially contradictory results, it seems, however, that an input characterized by maintaining the child’s attention using non-directive strategies, including verbal ones, and focused on the activity or object on which the child himself/herself is currently engaged has a positive influence on language skills, especially for high-risk children and at earlier ages (Hebert et al., 2004).

More recently Benassi et al. (2018) focused on a specific feature of parental linguistic input, considering maternal responses to babies’ communicative behaviors; the authors classified them according to their temporal contingency (i.e., whether they occurred within 5 s from the end of the infant’s communicative production) and the degree of relevance (i.e., whether they focused on the infant’s communicative behavior providing meaning to it). The data collected showed that maternal contingent relevant responses with a repeated label at 12 months were concurrently related to infants’ communication skills (i.e., pointing and giving gestures, words, and receptive communication) and predicted infants’ expressive communication skills at 24 months, even after controlling for neonatal status and 12-month expressive communication and cognitive skills.

Taken together, then, this empirical evidence supports the importance of considering the influence of both early infants’ abilities and quality of maternal input they receive for a deeper understanding of language development in preterm infants.

### Aims of the Study

Moving from the above considerations, the present study was designed to achieve two primary goals. The first was to deepen the analysis of semantically contingent linguistic input addressed to children born very preterm and to compare it to that directed to full-term children.

As this type of investigation has usually considered children with typical development and, in some cases, those with language delay, the identification of any similarities and/or differences in the strategies adopted by the two groups of mothers may extend our knowledge about the communicative environment to which preterm infants are exposed to. Since it is possible to hypothesize that these infants show a lower level of communicative-linguistic development, or at least a different repertoire of communicative behaviors, than full-term infants, the interaction strategies that mothers use to encourage their verbal spontaneous participation may also differ from those commonly adopted with typically developing children.

The second goal was to investigate synchronic interrelations between maternal semantically contingent utterances and infant’s communicative and linguistic skills in each of the two groups of dyads. From the literature, positive associations between maternal utterances and child language productivity are expected. However, assuming that the potential vulnerability of preterm infants makes them particularly susceptible to environmental factors, it is reasonable to hypothesize the presence of a pattern of associations that may be different from that found in full-term dyads. In addition, this examination can also help confirm the validity of some parental language strategies in supporting the communicative-language development of these children, leading to the identification of those that are most effective.

## Materials and Methods

### Participants

Thirty-six monolingual Italian mothers and their children participated in the study, including 16 very preterm infants (VPT; eight females) recruited at two neonatology follow-up services in Milan, and the remaining 20 born at term (FT; 10 females; groups did not differ for gender, *χ*^2^ = 0.00, *p* = 0.631) enrolled through notices posted in pediatric clinics in the same town. The main criteria for the selection of the very preterm children were a birthweight of less than 2000 grams (*M* = 1333.75, *SD* = 338.35), a gestational age under 32 weeks (*M* = 29.94, *SD* = 2.25) and the absence of genetic abnormalities, severe neurofunctional impairment, and/or sensorineural disabilities. Thus, the preterm children can be considered at low risk nevertheless their degree of prematurity. All full-term children were healthy and typically developing. Children born very preterm were mostly firstborn (VPT; 15 firstborn), while full-term children were more equally distributed between first and second born (FT; 11 firstborn; groups differed significantly for birth order, *χ*^2^ = 6.65, *p* = 0.010). Three very preterm participants were twins from different set of twins. Children in the very preterm and full-term groups did not differ for their mothers’ level of education that was overall medium-high (*χ*^2^ = 0.90, *p* = 0.710).

Each dyad was observed at children’s 24 months of age (VPT corrected age: *M* = 24.16 months, *SD* = 12.35 days, range = 23.50–24.97 months; chronological age: *M* = 26.16 months, *SD* = 18.01 days, range = 25.51–27.65 months; FT: *M* = 24.07 months, *SD* = 7.82 days; range: 23.53–24.53 months; *U* = 139.50, *p* = 0.519) and mothers were asked to fill out the questionnaire Primo Vocabolario del Bambino—scheda Parole e Frasi (Caselli and Casadio, 1995) which is the Italian version of the MacArthur-Bates Communicative Development Inventories—Words and Sentences form (Fenson et al., 1993, 2007). Data collected showed that the productive vocabulary size of VPT children (*M* = 179.54; *SD* = 192.62) was significantly lower than FT (*M* = 366.95; *SD* = 134.96), as attested by the statistical comparison carried out on the respective average values (*U* = 200.50; *p* = 0.009).

The study met ethical guidelines for human subject protections, including adherence to the legal requirements of Italy, and received formal approval by the local Research Ethical Committee of the University of Milano - Bicocca. All parents were informed about both the research procedure and general aims and gave informed written consent for study participation, data analysis, and data publication.

### Procedure

All mother-child dyads participated in a video-recorded semi-structured play session lasting approximately 20 min. VPT dyads were observed in a quiet room designed for observation at each of the two follow-up services involved, while full-term children and their mothers attended the session in the Early Childhood Observation Laboratory of the University of Milano - Bicocca.

In both observational contexts, which can be considered structurally equivalent, mothers and children sat together on a mat and mothers were encouraged to interact and play with their children freely as they would at home. The play materials have been selected with the specific goal of stimulating communicative and interactive exchanges in the dyads and consist of a series of age-appropriate toys (i.e., a toy farm with miniature people and animals, a telephone toy, a doll with clothes, and a kitchen set with pretend fruit and vegetables) and picture books. Each one was made available to mothers and children according to a fixed sequential order and it was not removed from the play area to ensure that each dyad had the opportunity to play with the toys that were most likely to stimulate their exchanges.

#### Coding and Measures

Maternal linguistic input and all spontaneous children’s communicative behaviors recorded during the observation sessions were entirely transcribed in CHAT format (CHILDES system; MacWhinney, 2000) by a trained observer, organizing them into separate utterances defined as any sequences of words, and/or prelinguistic sounds, and/or gestures preceded or followed by an auditory pause (1 s or more of non-speech), a change in the conversational turn, or an understandable modification in the intonation pattern.

Unclear speech in the transcriptions was reviewed by a second observer and resolved by checking the video-recorded material again.

##### Maternal Speech

From the transcripts, all maternal utterances that matched the ongoing topic of dyadic interaction were preliminarily identified as semantically contingent if: (a) they were produced in a joint attention situation and included a content word and/or (b) they were both temporally contiguous and linguistically and content-wise aligned to an infant production.

These utterances were, then, classified by means of a coding system developed by Girolametto et al. (2002) and partially modified for the aims of this study, which includes the following categories:

- Responsive labels: utterances that reflect the child’s focus of attention and highlight a content word referring to people, events, and objects (e.g., the mother says: *“C’è un cagnolino lì!”/“There’s a little dog there!”* while the child is looking at a picture book; *“Quanti frutti ci sono nel cesto!”/“So many fruits in the basket!,”* while the child is exploring a play food set).
- Interpretations: labels or short utterances aiming to disambiguate a child vocalization that is not clearly recognizable as a word, but it is produced with a communicative intention (e.g., the child vocalizes opening his arms and the mother says: *“È caduto!”/“It fell down!”*; the child vocalizes and points, and the mother says: *“Un pomodoro per il sugo”/“A tomato for the sauce”*).
- Reformulations: utterances that reproduce the child verbal production in a correct phonological form (e.g., the child says *“a falalla”* and the mother replies *“la farfalla”/“the butterfly”*; the child says *“etto è piccoino”* and the mother replies *“questo è piccolino”/“this is tiny”*).
- Imitations: partial or complete reproductions of the child’s preceding preverbal or verbal production (e.g., the child says *“palla blu”/“blue ball”* and the mother repeats *“palla blu”/“blue ball”*; the child says *“gira molto veloce”/“it turns very fast”* and the mother repeats *“gira veloce”/“it run fast”*).
- Expansions: utterances containing the repetition of an immediately preceding child’s verbalization and the addition of one or more morphemes or words (e.g., the child says *“un cappello”/“a hat”* and the mother replies *“un cappello come quello del nonno”/“a hat like grandpa’s”*; the child says *“è fiore”/“it’s flower”* and the mother repeats *“è un fiore”/“it’s a flower”*).

The following measures were then calculated: (a) the frequency per minute of utterances (as an index of speech productivity); (b) the proportion of semantically contingent utterances out of the total number of utterances. The proportions associated with each type of semantically contingent utterances were also computed. However, because children vary not only in the total number of spontaneous communicative behaviors they produce but also in their quality, mothers’ opportunities to respond contingently to children’s productions by using the various strategies considered also vary. For this reason, the proportional values were calculated by varying the denominator according to the meaning of each category of maternal contingent utterances. Consequently, the following measures were considered: (c) responsive labels on the total number of maternal utterances; (d) interpretations on the total number of children’s preverbal productions; (e) reformulations on the total number of children’s verbal productions; (f) imitations, and (g) expansions on the total number of children’s productions.

##### Children’s Prelinguistic and Verbal Behavior

In order to assess children’s prelinguistic and verbal repertoire, each transcribed utterance, excluding crying vocalizations and sounds of distress, was classified according to its complexity. Therefore, the following categories were considered:

- Preverbal productions: vocalizations containing a vowel or a syllable composed of a glottal or a glide consonant, single-syllable speech sounds, reduplicated and variegated babbling, and onomatopoeic sounds (e.g., “*ba,” “dada,” “bati,”/“bu-bu”*; Stoel-Gammon, 1989).
- One-word utterances: verbal productions consistently used to signal the same meaning and that approximate the sound of the conventional word used by adults (e.g., *“cappe” [scarpe]*/ *“shoes”*; *“bello,”*/*“beautiful”*; Vihman and McCune, 1994).
- Transitional forms: utterances composed of two or more vocal elements, in which at least one is a word, but that cannot be considered true multi-word utterances, including: content words anticipated by a single not meaningful vocalic element (e.g., *“/e/ fiore”/“/e/ flower”*); chained words, which are two words uttered with close temporal contiguity (less than 1 s) and which perform two separate speech acts (e.g., the child sees two figures on a book and says *“cane, gatto”/“dog, cat”*); horizontal repetitions, in which the child produces a word and repeats it in close temporal contiguity within the same turn (e.g., *“casa, casa,” “home, home”*; *“alto, alto,”*/*“tall, tall”*); frozen phrases, in which a number of words are uttered as a single word, since the distinct words are not used in other occurrences (e.g., the child uses the expression *“chi è?”/*“*who’s it?*,” but the function word *“chi”/“who,”* is produced only in this context); and non-word combinations, in which a content word is anticipated or followed by a multi-syllabic form not recognizable as a word in the adult lexicon (e.g., *“bimbo bedi”/“baby bedi,”* where *“bedi”* is a non-word; Fasolo and D’Odorico, 2012).
- Word combinations: utterances consisting of two or more words semantically and prosodically related to each other and uttered in a close temporal succession (e.g., *“questo latte” “this milk”*; *“voglio io” “I want”*; *“io siedo qui”*/*“I sit here”*; D’Odorico and Carubbi, 2003).

For each category, the proportional frequency was calculated out of the total number of utterances. The number of child utterances per minute was also computed as a measure of child talkativeness.

Interrater reliability was calculated using the intraclass correlation coefficient (ICC) obtaining high levels of agreement for all maternal (ICC: ≥0.95) and children measures (ICC: ≥0.96).

## Results

### Statistical Analyses

IBM SPSS Statistics 25 was used to conduct data analyses. Tests were bilateral with a statistical significance set at 0.05. Preliminary analyses have been performed to assess data distribution for normality. Results showed that most of the variables were not normally distributed with Kolmogorov-Smirnov and Shapiro-Wilk tests reporting *ps* < 0.01. For this reason, non-parametric tests were used.

A first set of Mann-Whitney tests was carried out to assess our first aim regarding the presence of differences in semantic contingency in mothers of very preterm and full-term children.

Secondly, we used Spearman correlations to explore the concurrent associations among maternal input variables and children’s linguistic measures, separately for very preterm and full-term participants. Before performing these analyses, a set of Mann-Whitney tests were performed to assess differences between very preterm and full-term children’s speech production.

### Semantic Contingency of Maternal Verbal Input in Very Preterm and Full-Term Mother-Child Dyads

The descriptive statistics for the measures of maternal input for all participants, and for mothers of very preterm and full-term children, are reported in Table 1. During the mother-child interactive session, mothers addressed their children with approximately 15 utterances per minute, with no difference being observed between mothers of very preterm and full-term children. 26% of maternal utterances were semantically contingent to children’s communicative initiatives, with mothers of full-term children using such utterances significantly more than mothers in the very preterm group. Furthermore, results indicated that mothers of full-term children also produced a significantly greater proportion of responsive labels over the total utterances produced. The same pattern emerged regarding the use of expansions, as full-terms mothers were observed to expand their children’s verbal productions more than mothers of very preterm children. Concerning the use of interpretations, imitations, and reformulations no significant differences due to birth condition were observed (Mann-Whitney test results are also reported in Table 1).

**Table 1:** Descriptive data for maternal speech input measures and Mann-Whitney tests comparing mothers of very preterm and full-term children.

	All participants (*n* = 36)			Very preterm group (*n* = 16)			Full-term group (*n* = 20)			*U*	*p*
	*M*	*SD*	Range	*M*	*SD*	Range	*M*	*SD*	Range		
Total Utterances per Minute	15.54	3.89	8.50–23.64	16.35	4.83	8.90–23.64	14.89	2.91	8.50–19.66	134.00	0.408
Contingent Utterances	0.26	0.08	0.05–0.38	0.21	0.07	0.05–0.34	0.30	0.06	0.18–0.38	48.50	<0.001
Responsive Labels	0.15	0.06	0.05–0.32	0.13	0.05	0.05–0.21	0.17	0.06	0.06–0.32	97.00	0.045
Interpretations	0.05	0.09	0.00–0.30	0.06	0.11	0.00–0.30	0.04	0.07	0.00–0.30	149.00	0.708
Imitations	0.09	0.10	0.00–0.51	0.11	0.14	0.00–0.51	0.07	0.05	0.01–0.17	157.00	0.924
Expansions	0.12	0.07	0.00–0.27	0.08	0.06	0.00–0.17	0.14	0.07	0.03–0.27	94.00	0.036
Reformulations	0.08	0.06	0.00–0.24	0.08	0.08	0.00–0.24	0.07	0.05	0.01–0.17	160.00	1.000

### Associations Between Input Semantic Contingency Measures and Children’s Speech Production

Table 2 summarizes the descriptive statistics of children’s speech measures collected during mother-child interactive sessions, for all participants and for each group separately. At a descriptive level, children produced almost six communicative productions per minute with no differences between very preterm and full-term participants. The communicative repertoires of very preterm children were mostly constituted by preverbal productions, followed by one-word utterances, transitional and combinational forms. Contrariwise, full-term children exhibited mostly verbal utterances. Comparing very preterm and full-term children, results showed that children born preterm exhibited more preverbal production and less transitional forms and word combinations than their peers born at term (Mann-Whitney test results are also reported in Table 2).

**Table 2:** Descriptive data for children’s speech measures and Mann-Whitney tests comparing mothers of very preterm and full-term children.

	All participants (*n* = 36)			Very preterm group (*n* = 16)			Full-term group (*n* = 20)			*U*	*p*
	*M*	*SD*	Range	*M*	*SD*	Range	*M*	*SD*	Range		
Productions per Minute	5.86	3.39	0.00–13.05	4.63	3.4	0.00–11.04	6.83	3.13	0.00–13.05	107.5	0.090
Preverbal Productions	0.35	0.27	0.07–1.00	0.59	0.24	0.28–1.00	0.17	0.08	0.07–0.33	2.50	<0.001
One-word Utterances	0.36	0.17	0.00–0.78	0.29	0.17	0.00–0.53	0.42	0.16	0.22–0.78	108.00	0.102
Transitional Forms	0.15	0.10	0.00–0.41	0.09	0.07	0.00–0.21	0.20	0.09	0.03–0.41	53.50	<0.001
Word Combinations	0.13	0.13	0.00–0.42	0.03	0.04	0.00–0.14	0.21	0.13	0.02–0.42	26.00	<0.001

Results of correlational analyses carried out to assess synchronic associations between maternal speech measures and children’s communicative repertoire variables within each group are shown in Table 3. Maternal use of semantically contingent utterances was associated with better children’s outcomes both in very preterm and full-term children. As a matter of fact, contingent utterances correlated negatively with children’s use of preverbal productions in both groups, and positively with one-word, transitional and combinational utterances in very preterm children, and with word combinations in full-term participants. Moreover, mothers’ use of expansions of children’s productions was significantly and positively associated with the use of one-word and transitional utterances in the very preterm group and, similarly, to the use of one-word utterances in children born at term. Maternal expansions were also negatively associated with the amount of preverbal productions in very preterm children. Finally, mothers’ use of imitations was observed as positively associated with very preterm children’s use of one-word utterances and negatively with their preverbal productions.

**Table 3:** Results of Spearman correlation performed between speech input measures and children’s speech measures for the very preterm and the full-term group.

	Contingent utterances	Responsive labels	Interpretations	Imitations	Expansions	Reformulations
**Very Preterm Children**						
Preverbal Productions	−0.635^**^	−0.135	0.090	−0.634^**^	−0.700^**^	−0.350
One-word Utterances	0.588^*^	0.018	0.209	0.815^**^	0.768^**^	0.370
Transitional Forms	0.519^*^	0.094	−0.224	0.280	0.522^*^	0.415
Word Combinations	0.543^*^	0.132	−0.441	0.366	0.353	0.414
**Full-term Children**						
Preverbal Productions	−0.546^*^	−0.269	0.111	0.006	0.066	0.080
One-word Utterances	−0.146	−0.223	0.370	−0.320	0.466^*^	0.414
Transitional Forms	−0.081	−0.011	−0.411	0.250	−0.280	−0.292
Word Combinations	0.647^**^	0.346	−0.265	0.119	−0.242	−0.421

* *p* < 0.05;

** *p* < 0.01.

## Discussion

This observational study represents one of the first attempts to examine maternal semantically contingent input directed to very preterm children. More specifically, the first aim was to investigate whether mothers differed in the verbal strategies they adopted in response both to their children focus of attention and spontaneous utterances depending on birth condition.

Overall, the results showed that, although mothers of both groups exhibited similar verbal productivity, those of very preterm children produced proportionally fewer semantically contingent utterances during interactions, compared to full-term mothers. Thus, even though the two groups of children were exposed to a similar maternal amount of maternal language, the linguistic input directed at very preterm children appeared to match to a lesser extent the ongoing topic of the interaction. Furthermore, this maternal interactive style did not appear to depend directly on children’s talkativeness, as no significant difference emerged between the two groups of children in the frequency of utterances per minute. This finding seems to be in line with some studies showing that mothers of preterm infants appear to be less able to establish an interactive symmetry, especially documented in the early stages of development. On this matter, mothers of preterm infants have been described as more intrusive and less synchronous during social exchanges with their infants, demonstrating poor competence in coordinating their interactive behaviors with the infant’s alertness (Greenberg and Crnic, 1988; Feldman and Eidelman, 2007; Salerni et al., 2007; Provasi, 2019).

However, very preterm children participating in the study, regardless of their verbal productivity, showed a different spontaneous communicative-linguistic repertoire than term children, mainly characterized by preverbal productions, while transitional forms and word combinations were less frequently produced. Therefore, their less advanced language skills may have affected the mothers’ opportunities to use some specific types of semantically contingent utterances in a relevant way. In fact, if we consider the various categories of semantically contingent utterances controlling, when appropriate, for the communicative and linguistic behaviors spontaneously produced by children, the only differences between the two groups of mothers are those referring to responsive labels and expansions. Specifically, mothers of preterm infants were less likely to provide verbal information that were semantically related to their children attentional focus and to follow infants’ preverbal or verbal productions by repeating them and adding syntactic or semantic information. Taken together, these findings suggest that the language environment to which preterm infants are exposed to has certain characteristics that make it less optimal for language development, as evidenced by studies that have found positive associations between measures of maternal semantically contingent language and infant language outcomes (Tamis-LeMonda et al., 2001, 2014; Vigil et al., 2005; Roseberry et al., 2014; Masek et al., 2021).

A similar pattern of relationships also emerged in the present study, in that mothers’ greater use of semantically contingent utterances corresponds to a children’s more advanced communicative-linguistic repertoire. In both very preterm and full-term children, this sort of maternal input is concurrently related to a lower spontaneous production of preverbal communicative behaviors and to a greater use of combinatorial utterances. Moreover, positive associations were found, in very preterm dyads only, between maternal semantically contingent utterances and one-word and transitional forms produced by children during interaction.

From the observed pattern of correlations, a particular role in this type of interrelations seems to be played by maternal utterances aimed at expanding children’s productions and, limited to very preterm children, imitating them, a result, the latter, in line with that found for Italian late talkers (Girolametto et al., 2002; Suttora et al., 2021). Both these linguistic strategies emphasize the communicative value of the child’s vocal/verbal productions and, at the same time, lead the child to focus his/her attention on a new stimulus inserted in an immediate and familiar linguistic context, thus facilitating the extraction of relevant information and enhancing child language development (Girolametto et al., 1999).

The lack of associations found between maternal responsive labeling and child language measures deserves a special consideration. Such a finding appears to be in contrast with previous empirical evidence in which labels produced within joint attention episodes promoted vocabulary learning, both in typically developing children and in those born preterm (Goldin-Meadow et al., 2007; Olson and Masur, 2015; Benassi et al., 2018).

In this regard, it is possible to hypothesize that in the developmental stage considered in this study, expressive language skills are more supported by contingent maternal responses to child productions, rather than by the simple exposure to the naming of objects or events that fall within the child’s attentional focus. In other words, although labeling may be considered a relevant strategy in the period when children need to learn the first vocabulary, its effectiveness may be reduced when the stage-specific task is to expand the productive vocabulary and verbally express semantic relations. In this case, children may benefit from a richer linguistic input.

Taken together, the results of this study provide evidence for an effect of mothers’ semantically contingent input on the variability of linguistic competences of children who are expanding their expressive lexicon and acquiring early combinatorial skills, confirming previous studies conducted with typically developing children and extending this evidence to children born very preterm (Tamis-LeMonda et al., 2001; Masek et al., 2021). From a theoretical point of view, then, this study supports a perspective of language acquisition that relies on an interaction between children’s processing mechanisms and the content of CDS they are exposed to.

In this sense, it is also important to acknowledge that the associations detected in this study do not necessarily represent direct influences of maternal input on the child’s language abilities. The results, in fact, can also be interpreted from the perspective of how the child influences the parent (Conti-Ramsden, 1990). Recent studies showed that very preterm infants are characterized by difficulties and delays in several areas of development, including psychomotor skills (Fuentefria et al., 2017), temperamental traits (Cassiano et al., 2020), and social-emotional competences (Yaari et al., 2018), which can impact on the overall quality of mother-infant interaction from early on. These possible sources of variation along with the continuous transactions, over time, between the child and his/her proximal environment might result in specific maternal behaviors and attitudes which reflected in different communicative and interactive styles.

At the same time, the pattern of highlighted associations suggests the relevance of considering maternal linguistic responsivity as a modular construct, as particular linguistic strategies may serve different functions and exert specific effects on children’s language at different stages of development (Bornstein et al., 2008). In this sense, considering maternal language responsiveness in a more differentiated way also allows for a better understanding of it in different populations, leading to the identification of specificities that would remain unnoticed if more global measures were used.

### Implication for Clinical Practice

The interventions directed at promoting language development in populations of children with language delay (Heidlage et al., 2020) and of children with secondary linguistic issues (Pennington and Thomson, 2007; Weitzman, 2013) have been proven to be effective in supporting children language development. One basic aim of such interventions is to enhance parents’ responsiveness, making their input much more attuned to their children’s focus of attention and responsive to their communicative bids. This research suggests that special attention should be paid to parents’ provision of semantically contingent linguistic input, beyond the importance that structural features of language may take on. Furthermore, the findings point at the input provided to very preterm children, encouraging clinicians to set up projects and interventions aimed at ameliorating their language environment.

### Limitations and Strengths of the Study

The study presents some limitations that should be considered. The first is that the sample is limited in terms of size which can lead to issues of generalization of the findings. However, given the peculiarity of the population in exam, i.e., very preterm children, the study should be considered exploratory in its aims. Another issue concerns the different distribution of firstborn children in the groups, with preterm children being mostly firstborn and full-term being equally distributed between first and laterborn. Even if we cannot rule out the effect of parity from the interpretation of our findings, previous literature reports that being firstborn represents a protective factor and not a risk for language development and for input exposure, as first-time mothers usually spend more quality time with their children than mothers of laterborn children. Findings documented that firstborns are often favored in their language development, showing an earlier lexical onset and greater lexical acquisition than laterborns (Hoff, 2003; Nafissi and Vosoughi, 2015). From these data, we can hypothesize that the lower level of semantically contingency found in mothers of very preterm children is most probably due to the peculiarity of this birth condition, rather than by parity, but still, new evidence is needed in support of this thesis.

Another limit that has to be acknowledged refers to the correlational design adopted which not allowed to disambiguate the direction of the associations between maternal and children’s measures. In this regard, longitudinal studies are needed to also investigate whether semantically contingent maternal input changes over time and to understand the different impact that specific indices may have at different developmental stages.

This study has some relevant strengths too. The first is represented by the detailed analysis of maternal and child speech performed, which provided rich information on the types of maternal input contingency and on the quality and quantity of children’s verbal utterances. Another point of strength is the choice of the target population of very preterm children. Literature highlights that children with higher degrees of immaturity, such as very preterm children, are at risk of developing a language delay (Sansavini et al., 2010, 2011); identifying those aspects that can support or rather further hinder their language development is pivotal for research and clinic.

## Conclusion

The present work contributes with new data to the understanding of the role of maternal verbal input contingency in children communicative and linguistic development, with a specific focus on the effects of preterm birth on this topic. Overall, the study highlights that mothers of children born very preterm differ from full-term in the way they respond to their children’s communicative bids and use their verbal input to attune to their children’s ongoing focus of interest. In addition, our findings reveal that a higher use of semantically contingent utterances is associated with children’s more mature communicative repertoires. These findings suggest the opportunity to address parental speech contingency to implement intervention targeted at foster communicative and language development in very preterm children.

## Data Availability Statement

The original contributions presented in the study are included in the article/supplementary files, and further inquiries can be directed to the corresponding author.

## Ethics Statement

The studies involving human participants were reviewed and approved by the Ethics Committee of University of Milano - Bicocca. Written informed consent to participate in this study was provided by the participants’ legal guardian/next of kin.

## Author Contributions

NS: conceptualization and methodology. NS and CS: data collection, data transcription, coding, analysis, writing original draft, review, and editing. All authors have read and approved the manuscript.

## Conflict of Interest

The authors declare that the research was conducted in the absence of any commercial or financial relationships that could be construed as a potential conflict of interest.

## Publisher’s Note

All claims expressed in this article are solely those of the authors and do not necessarily represent those of their affiliated organizations, or those of the publisher, the editors and the reviewers. Any product that may be evaluated in this article, or claim that may be made by its manufacturer, is not guaranteed or endorsed by the publisher.
